# Transcranial Focal Electric Stimulation Avoids P-Glycoprotein Over-Expression during Electrical Amygdala Kindling and Delays Epileptogenesis in Rats

**DOI:** 10.3390/life13061294

**Published:** 2023-05-31

**Authors:** Daniel Fonseca-Barriendos, José Luis Castañeda-Cabral, Frida Martínez-Cuevas, Walter Besio, Alejandro Valdés-Cruz, Luisa Rocha

**Affiliations:** 1 Departamento de Farmacobiología, Centro de Investigación y de Estudios Avanzados, Ciudad de México C.P. 14330, Mexicofridamartinezcuevas@cinvestav.mx (F.M.-C.); 2 Departamento de Biología Celular y Molecular, Centro Universitrio de Ciencias Biológicas y Agropecuaias, Universidad de Guadalajara, Zapopan C.P. 44600, Mexico; jose.castanedacabral@yahoo.com.mx; 3 Department of Electrical, Computer, and Biomedical Engineering, University of Rhode Island, Kingston, RI 028881, USA; besio@uri.edu; 4 Laboratorio de Neurofisiología del Control y la Regulación, Instituto Nacional de Psiquiatría “Ramón de la Fuente Muñiz”, Ciudad de México C.P. 14370, Mexico

**Keywords:** P-glycoprotein, epileptogenesis, neuromodulation, hippocampus, neocortex, kindling, TFS

## Abstract

Recent evidence suggests that P-glycoprotein (P-gp) overexpression mediates hyperexcitability and is associated with epileptogenesis. Transcranial focal electrical stimulation (TFS) delays epileptogenesis and inhibits P-gp overexpression after a generalized seizure. Here, first we measured P-gp expression during epileptogenesis and second, we assessed if TFS antiepileptogenic effect was related with P-gp overexpression avoidance. Male Wistar rats were implanted in right basolateral amygdala and stimulated daily for electrical amygdala kindling (EAK), P-gp expression was assessed during epileptogenesis in relevant brain areas. Stage I group showed 85% increase in P-gp in ipsilateral hippocampus (*p* < 0.001). Stage III group presented 58% and 57% increase in P-gp in both hippocampi (*p* < 0.05). Kindled group had 92% and 90% increase in P-gp in both hippocampi (*p* < 0.01), and 93% and 143% increase in both neocortices (*p* < 0.01). For the second experiment, TFS was administrated daily after each EAK stimulation for 20 days and P-gp concentration was assessed. No changes were found in the TFS group (*p* > 0.05). Kindled group showed 132% and 138% increase in P-gp in both hippocampi (*p* < 0.001) and 51% and 92% increase in both cortices (*p* < 0.001). Kindled + TFS group presented no changes (*p* > 0.05). Our experiments revealed that progression of EAK is associated with increased P-gp expression. These changes are structure-specific and dependent on seizure severity. EAK-induced P-gp overexpression would be associated with neuronal hyperexcitability and thus, epileptogenesis. P-gp could be a novel therapeutical target to avoid epileptogenesis. In accordance with this, TFS inhibited P-gp overexpression and interfered with EAK. An important limitation of the present study is that P-gp neuronal expression was not evaluated under the different experimental conditions. Future studies should be carried out to determine P-gp neuronal overexpression in hyperexcitable networks during epileptogenesis. The TFS-induced lessening of P-gp overexpression could be a novel therapeutical strategy to avoid epileptogenesis in high-risk patients.

## 1. Introduction

P-glycoprotein (P-gp) is a 170 KDa transmembrane protein member of the ABC transporter family [1]. Under physiological conditions, P-gp is expressed in several organs such as the colon, kidneys, liver, among others [2,3]. In the central nervous system, P-gp is expressed in the endothelial cells and astrocyte foot process of the blood brain barrier (BBB); and the choroid plexus [4,5,6,7,8]. P-gp expression in these cells has been related to protection of the brain parenchyma from cytotoxic damage [9,10]. However, P-gp overexpression in epilepsy has been associated with multidrug-resistant phenotype [11,12,13,14,15]. Aberrant expression of P-gp in neurons has been described in brain tissue resected from patients with drug-resistant epilepsy and an animal model of severe seizures [12,16,17,18]. P-gp overexpression may be induced by several mechanisms including hypoxia [19,20], neuroinflammation [21,22], and/or excessive glutamate release [22,23], all conditions associated with seizure activity [24,25,26,27,28].

On the other hand, transcranial focal electrical stimulation (TFS) via tripolar concentric ring electrodes (TCRE) is a novel noninvasive neuromodulation strategy. TFS focuses on the electrical stimulation, with a uniform current density, directly below the electrode generating electric fields in subcortical regions without adverse effects [29,30,31,32,33]. TFS reduce the convulsive activity induced by several drugs [34,35,36,37,38]. Santana-Gómez and colleagues found that TFS reduced the seizure activity and lessens the excessive glutamate release during pilocarpine-induced status epilepticus [39].

Meanwhile, Pérez-Pérez and colleagues found that TFS restitutes the anticonvulsive effects of phenytoin in an animal model of pharmacoresistant seizures with P-gp overexpression. In addition, these authors found that TFS applied before the induction of a single generalized seizure avoids P-gp overexpression in both hippocampi and cerebral cortices [38]. Concerning epileptogenesis, it was found that TFS applied immediately after each electrical amygdala kindling (EAK) stimulation avoids the evolution of this process in cats, and this effect lasted several weeks after TFS cessation [40].

For the present study, we suggest that the progressive augmentation of neuronal excitability during epileptogenesis correlates with gradual P-gp overexpression. This hypothesis is supported because P-gp expressed in neurons may alter the resting membrane potential as a consequence of changes in the volume-gated chloride channels, the cellular pH, and acting as a lipid flippase [41,42,43,44,45]. We also suggest that TFS avoids P-gp overexpression during epileptogenesis. Here, we aimed to assess P-gp expression through epileptogenesis induced by EAK in both hippocampi and frontal cortices, areas related to seizure propagation in the rat brain [46,47,48,49]. We also investigated if the antiepileptogenic effect of TFS avoids the EAK-induced P-gp overexpression.

## 2. Materials and Methods

### 2.1. Animals

Male Wistar rats (300–350 g body weight), individually housed and maintained under environmentally controlled conditions (12 h light/dark cycles, 22 °C) with food and water ad libitum were used in the present study. All experiments were approved by the ethics committee of the National Institute of Psychiatry Ramon de la Fuente Muñiz (project NC123240.1) and performed according to the Mexican law for the care and use of laboratory animals (NOM-062-ZOO-1999).

### 2.2. Experiment 1: Evaluation of P-gp Expression during EAK-Induced Epileptogenesis

Surgery was performed with a mixture of Zoletil 50 (50 mg/kg IM) and xylazine hydrochloride (12 mg/kg IM). Stainless steel tripolar electrodes, insulated except at the tip, were stereotaxically implanted in the right basolateral amygdala (AP-2.8, L + 4.8, V-8.5) [50]. Two tips of the tripolar electrodes were used for electrical stimulation and the third tip was used for signal recording. Two epidural electrodes were also placed in both prefrontal cortices for electrographic recording. Additionally, three ground screws were implanted. All electrodes and ground screws were soldered into one header and the entire preparation was fixed to the skull with dental acrylic. Rats were allowed to recover for 7–10 days before any manipulation and were treated with oral antibiotics (enrofloxacin 12 mg/kg).

#### 2.2.1. Experimental Groups

**Figure 1:**
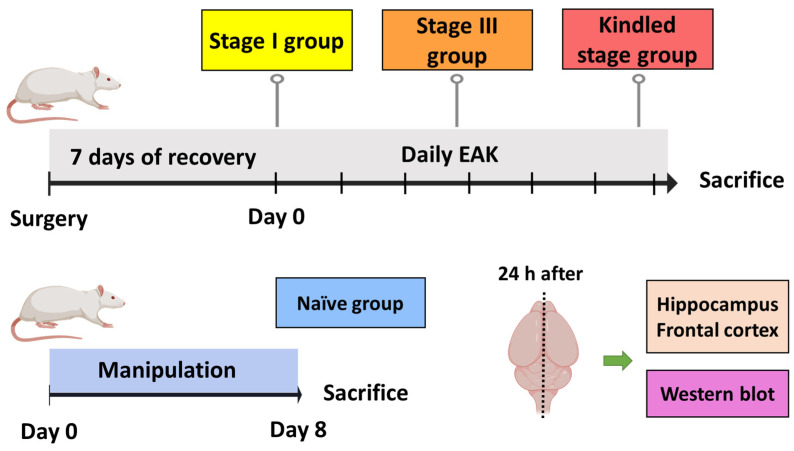
Experimental protocol designed to evaluate P-gp expression during EAK-induced epileptogenesis. The kindled group was stimulated daily until three consecutive stage V seizures were evoked. The stage III group was stimulated daily until the first stage III seizure was evoked. The stage I group only received the ADT determination. The naïve group was manipulated as the previous group, but no surgery nor electrical stimulation were performed. All animals were sacrificed 24 h after the last manipulation. The ipsi- and contralateral hippocampi and frontal cortices were obtained for subsequent estimation of protein expression by Western blot. *n* = 5 per group.

Kindled group (*n* = 5). After recovery, after-discharge threshold (ADT) was estimated. Then, daily EAK stimulation was applied until three consecutive stage V seizures were elicited. Animals were decapitated 24 h after the last stage V seizure and resection of the ipsi- and contralateral hippocampi and neocortices was performed. All tissue was stored at −70 °C until used to assess P-gp expression by Western blot (Figure 1).

Stage III group (*n* = 5). The experimental manipulation was as described above, except that animals were decapitated 24 h after the first stage III seizure.

Stage I group (*n* = 5). Animals were manipulated as described above, except that they were decapitated 24 h after the ADT estimation.

Naïve group (*n* = 5). Rats were handled as the stage I group. However, no surgery or electrical stimulation were performed. Results obtained from this group were considered as control condition.

#### 2.2.2. ADT, EAK, and EEG

Two tips of the tripolar electrode implanted in the right basolateral amygdala were used for ADT determination. The electrical stimulation (1 ms monophasic square pulses at 60 Hz for 1 s) started at 100 µA through a S11 stimulator (Grass, MA, USA). The current intensity was increased by 20% every 3 min until the animals presented ipsilateral eye blink accompanied by a brief afterdischarge (3–4 s) [51]. Daily EAK started the following day using the ADT parameters previously determined and behavioral assessment was performed according to the Racine’s scale [52]. Electrographic activity from the epidural and amygdala electrodes was recorded using a 78 E polygraphic equipment (Grass, MA, USA), preamplified and band-pass filtered between 3 and 300 Hz, and acquired on-line (500 Hz sampling frequency) using an analog to digital conversion system (ADQ8CH). Signal analysis was conducted off-line using a computational program (ADQ8CH, Mexico City, Mexico). Both the analog to digital conversion system and the software were custom made [40,53]. All signal analysis was performed offline.

#### 2.2.3. Western Blotting

Western blot is a sensitive semi quantitative technique that allows us to identify changes in protein expression even if they are expressed in picograms. With proper antibodies, this technique is highly sensitive and specific and thus, was chosen to assess P-gp expression during EAK-induced epileptogenesis [54].

Brain samples were homogenized in lysis buffer (50 mM Tris-HCl pH 7.5, 150 mM NaCl, 1 mM EDTA, and 0.1% Triton X-100) with a protease inhibitor cocktail (Roche Diagnostics GmbH, Germany) in a cold bath at 4 °C. Then, homogenates were centrifuged at 14,000× *g* for 10 min at 4 °C and the supernatant (total protein extract) was immediately collected, aliquoted, and frozen at 70 °C until use. Protein concentration was assessed in the extracts according to the Bradford method (Bio-Rad Laboratories, Hercules, CA, USA) using bovine serum albumin (BSA) (Bio-Rad Laboratories, Hercules, CA, USA) as standard. Samples (50 μg) of total protein extract were boiled for 5 min at 95 °C in Laemmli buffer (500 mM Tris-HCl pH 6.8, 2% SDS, 10% glycerol, 10% β-mercaptoethanol, and 0.1% bromophenol blue). Electrophoresis was carried out in running buffer (25 mM Tris, 192 mM glycine, and 0.1% SDS, pH 8.3; Bio-Rad Laboratories, Hercules, CA, USA) at 85 V for 30 min and 100 V for 2 h SDS-polyacrylamide gel electrophoresis (7.5%). The electrotransference was done on a polyvinylidene difluoride membrane (Immun-Blot, Bio-Rad Laboratories, Hercules, CA, USA) at 110 V for 30 min using transfer buffer (25 mM Trizma base, 250 mM glycine, and 20% methanol, pH 8.3). Unspecific binding was blocked for 1 h at 4 °C with a 5% blocking solution (Blot-QuickBlocker, EMD Millipore, Oakville, Ontario, CA, USA) in TBS-T buffer (20 mM Tris, 500 mM NaCl, 0.1% Tween 20, pH 7.5). The membranes were incubated overnight with the primary antibodies at 4 °C with gentle shaking, used as follows: rabbit monoclonal anti-P-gp (1:1000; Cat. ab170904; Abcam, Waltham, MA, USA) and rabbit monoclonal anti-actin (1:1000; Cat. ab179467; Abcam, Walthman, MA, USA). All primary antibodies were diluted in TBS buffer (20 mM Tris, 500 mM NaCl, pH 7.5). Membranes were washed 3× with TBS-T buffer at 4 °C for 5 min each and followed by incubation with the corresponding secondary antibody HRP-goat anti-rabbit IgG (1:5000 and 1:10,000 for P-gp and actin, respectively) for 2 h at 4 °C diluted in TBS. Finally, the membranes were incubated in peroxide/luminol solution (Clarity Western ECL substrate, Bio-Rad Laboratories, Hercules, CA, USA) at room temperature for 5 min. The chemiluminescent data were normalized using actin as constitutive protein, resulting in an expression ratio relative, with determinations by duplicate.

### 2.3. Experiment 2: Evaluation of TFS on the EAK-Induced P-gp Overexpression

This experiment was designed to assess if the antiepileptogenic effect of TFS avoids the EAK-induced P-gp overexpression.

#### Experimental Groups

**Figure 2:**
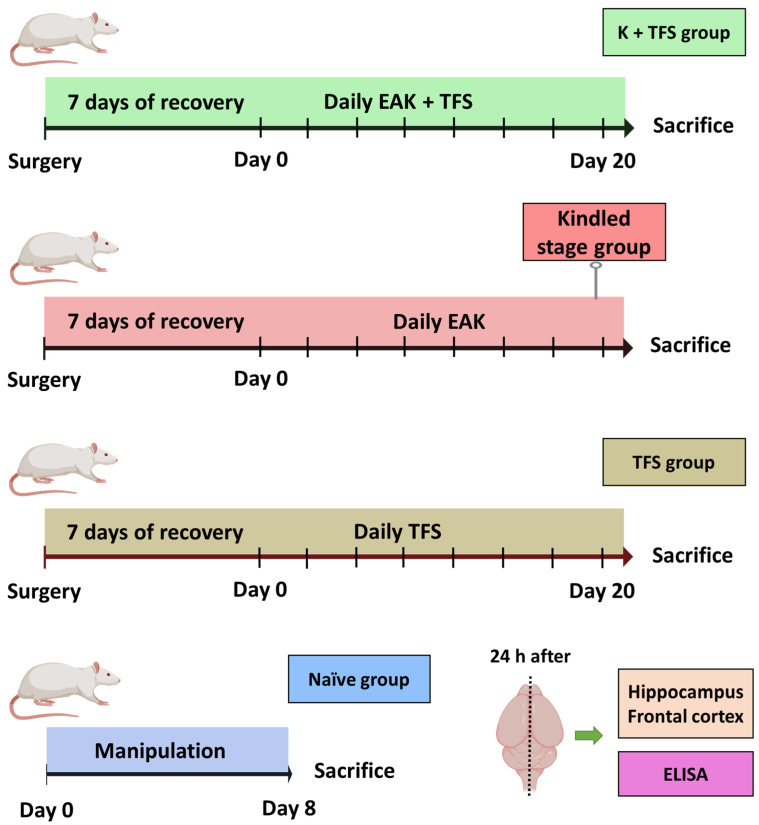
Experimental protocol designed to evaluate the effect of TFS on the EAK-induced P-gp overexpression. The kindled + TFS group (K + TFS) received daily kindling stimulation immediately followed by TFS for 20 days. The kindled group was stimulated daily until three consecutive stage V seizures were evoked. The TFS group received daily TFS for 20 days. The naïve group was manipulated as the previous groups, but no surgery nor electrical stimulation were performed. Animals were sacrificed 24 h after the last manipulation. The ipsi- and contralateral hippocampi and frontal cortices were obtained for subsequent estimation of protein expression by Elisa assay. *n* = 5 per group.

Kindled + TFS (K + TFS) (*n* = 5). Surgery was performed as described previously (Section 2.2) except that a TCRE (6 mm diameter) was placed over the sagittal suture, between bregma and lambda. Electrodes were soldered into two headers: one for TFS and the rest for EAK, including grounds. ADT was estimated as described in Section 2.2.2. Thereafter, rats received EAK and immediately after, TFS was applied for 2 min (300 Hz, 200 µs biphasic square pulses at 1.5 mA) through the outer ring and the central disc (with the middle ring floating) of the TCRE using a S88 stimulator (Grass, MA, USA). This procedure was repeated daily for 20 days. Behavioral and EEG signals were recorded during EAK and TFS and were analyzed as described in Section 2.2.2. Rats were decapitated 24 h after the last electrical stimulation and resection of hippocampi and neocortices (ipsi- and contralateral) was performed (Figure 2). P-gp protein concentration was evaluated by Elisa assay following the manufacturer’s instructions (US Biological Life Sciences Cat. 353596). An Elisa assay was chosen for this experiment as this quantitative technique has high sensitivity and yields high throughput results [55].

Kindled (*n* = 5). Animals were handled as described for the K + TFS group, except that TFS was not applied. Stimulation was stopped when rats presented three consecutive stage V seizures and tissue collection was performed as described above.

TFS (*n* = 5). Animals were handled as described for the K + TFS group, except that only TFS was applied daily for 20 days.

Naïve (*n* = 5). Rats were handled as the TFS group. However, no surgery or electrical stimulation were performed. Results of this group were considered as control condition.

### 2.4. Statistical Analysis

EAK progression, Western blot and Elisa assay data are expressed as mean ± standard deviation. Normality was assessed by Shapiro–Wilk test. Statistical analysis of group differences was performed by one way analysis of variance (ANOVA), two-way ANOVA for repeated measures followed by Tukey post hoc test and unpaired T test. Spearman’s correlation analysis was performed between EAK data and Western blot/Elisa results. Significance level was set to *p* < 0.05. All statistical analysis and graph construction were performed using GraphPad Prism Software v.8.01 (GraphPad Software Inc., Boston, MA, USA).

## 3. Results

### 3.1. EAK-Induced Epileptogenesis Is Associated with P-gp Overexpression

**Figure 3:**
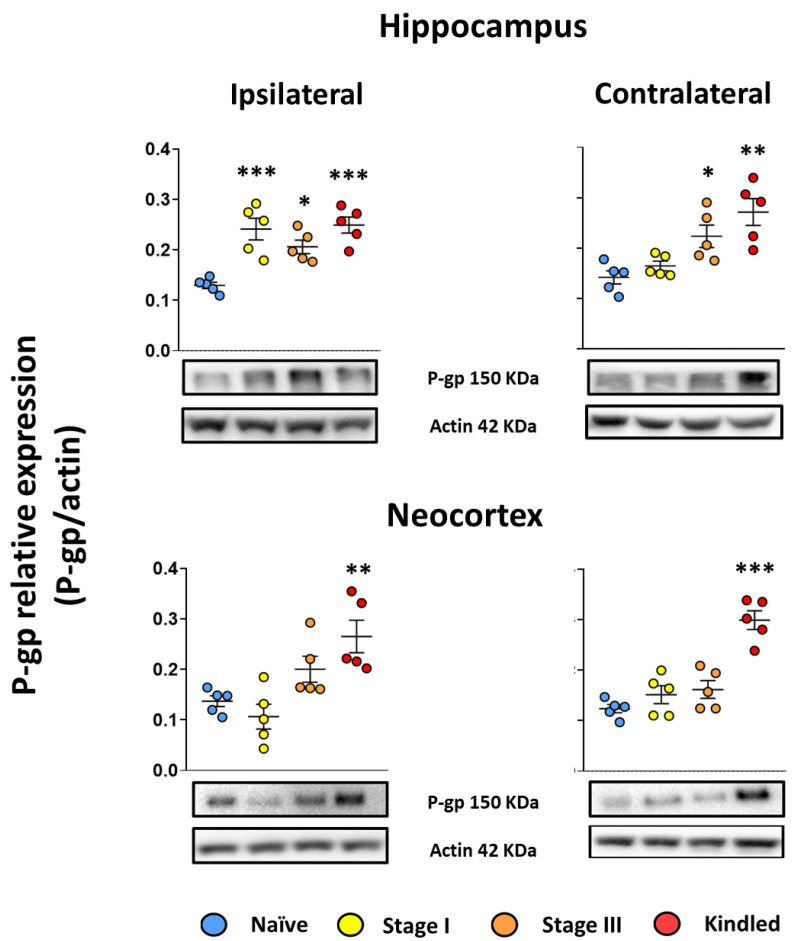
EAK-induced epileptogenesis is associated with P-gp overexpression. Graph represents the expression ratio of P-gp relative to actin in the hippocampus (**upper** panels) and neocortex (**lower** panels) ipsi- (**left** panels) and contralateral (**right** panels) to the amygdala stimulated of stage I, stage III, and Kindled groups. Naïve data represent right and left structures as no surgery was performed. Representative images of P-gp detection and its respective loading control protein actin are included. Data show the mean ± SEM. Each sample was assessed by duplicate. Statistical significance between groups is represented by * *p* < 0.05, ** *p* < 0.01, and *** *p* < 0.001. ANOVA test with Tukey’s multiple comparisons test.

The naïve group did not present any behavioral modification during handling. Western blot results showed that the hippocampal P-gp relative expression (P-gp/actin) was 0.12 ± 0.014 and 0.14 ± 0.03 right and left, respectively. As for the neocortex, the P-gp relative expression index was 0.13 ± 0.023 and 0.12 ± 0.018 right and left, respectively (Figure 3).

Concerning the stage I group, the ADT was 284 ± 92 µA. During the ADT estimation, the amygdala EEG recordings revealed an afterdischarge of 3.11 ± 1.7 s duration with a spike frequency of 1.3 ± 0.15 spikes per second. No epileptiform activity was recorded in the epidural electrodes. Western blot analysis revealed an 85% increased relative expression of P-gp in hippocampus ipsilateral to the amygdala stimulated (0.24 ± 0.048, *p* < 0.001 vs. naïve group). No changes were found in the contralateral hippocampus (0.16 ± 0.02, *p* > 0.05 vs. naïve group) nor the neocortices (0.10 ± 0.054 and 0.15 ± 0.04 ipsi- and contralateral, respectively, both *p* > 0.05 vs. naïve) (Figure 3). Correlation analysis was performed with the EEG data (afterdischarge duration and spike frequency), ADT and P-gp relative expression; however, no significant correlations were found. 

The ADT for the stage III group was 341 ± 132 µA (*p* > 0.05 vs. stage I group) and 4 ± 1 kindling trials were necessary to achieve the first stage III seizure. The first stage III seizure was associated with an afterdischarge in the amygdala stimulated lasting for 13.31 ± 7.14 s (*p* > 0.05 vs. stage I group) and with a spike frequency of 2.13 ± 0.25 (*p* < 0.001 vs. stage I group). Electrographic recording revealed an afterdischarge propagation to the cortex (13.1 ± 6.68 s of duration with a spike frequency of 1.67 ± 0.76). Western blot analysis revealed a 58.9% and 57.14% increase in P-gp expression in both hippocampi (0.20 + 0.03 and 0.22 ± 0.049 ipsi- and contralateral to the amygdala stimulated, respectively, both *p* < 0.05 vs. naïve group; *p* > 0.05 vs. stage I group ipsilateral hippocampus). No changes were found in the neocortices (0.2 ± 0.057 and 0.16 ± 0.039, ipsi- and contralateral respectively, *p* > 0.05 vs. naïve group) (Figure 3). Correlation analysis was performed with the EEG data (afterdischarge duration and spike frequency), ADT, number of kindling trials, and p-gp relative expression; however, no significant correlations were found.

The ADT for the Kindled group was 446 ± 125 µA (*p* > 0.05 vs. stage I and stage III groups). A total of 15 ± 1 kindling trials were applied to achieve the kindled stage. During the last stage V seizure, the EEG recording revealed an afterdischarge of 75.29 ± 44.63 s of duration (*p* < 0.01 vs. stage III group and *p* < 0.001 vs. stage I group) with a spike frequency of 2.26 ± 0.07 (*p* < 0.001 vs. stage I group and *p* > 0.05 vs. stage III group). The afterdischarge propagated to the cortex and lasted 74 ± 42.43 s (*p* < 0.05 vs. stage III group) with a frequency of 2.2 ± 0.11 (*p* > 0.05 vs. stage III group). Western blot analysis revealed a 92% and 90% increase in p-gp expression in both hippocampi (0.249 ± 0.035 and 0.271 ± 0.06, *p* < 0.001 and *p* < 0.01 vs. naïve group, ipsi- and contralateral to the stimulated amygdala, respectively). As for the neocortices, a 93% and 143% increase in P-gp relative expression was found (0.26 ± 0.07 and 0.299 ± 0.04, *p* < 0.01 and *p* < 0.001 vs. naïve group, ipsi- and contralateral, respectively) (Figure 3). Correlation analysis revealed that higher spike frequency during stage V seizures correlated with higher P-gp protein expression in the contralateral hippocampus (Table 1).

### 3.2. TFS Avoids P-gp Overexpression during the EAK-Induced Epileptogenesis

The naïve group did not present any behavioral alterations. Elisa assay revealed a P-gp protein concentration of 48.05 ± 12.61 ng/mL in the right hippocampus, 39.99 ± 8.63 ng/mL in the left hippocampus, 66.03 ± 15.27 ng/mL in the right neocortex, and 50.47 ± 13.03 ng/mL in the left neocortex (Figure 2).

**Figure 4:**
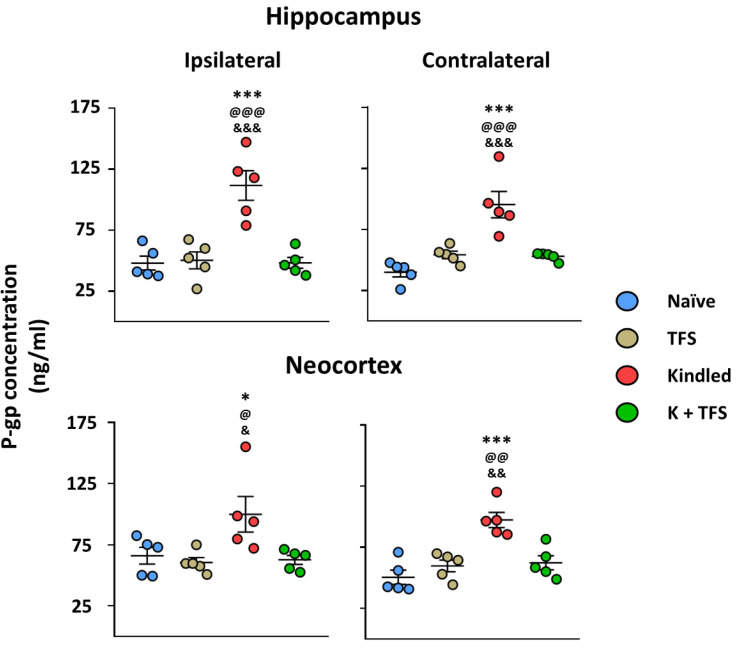
TFS avoids P-gp overexpression during the EAK-induced epileptogenesis. Graph represents the P-gp protein concentration in the hippocampus (**upper** panels) and neocortex (**lower** panels) ipsi- (**left** panels) and contralateral (**right** panels) to the amygdala stimulated of Kindled and K + TFS groups. Naïve data represent right and left structures as no surgery was performed. TFS data represent ipsi- and contralateral of the amygdala implanted. Data show the mean ± SEM. Each sample was assessed by duplicate. Statistical significance between groups is represented by * *p* < 0.05 and *** *p* < 0.001 vs. naïve group; ^@^ *p* < 0.05, ^@@^ *p* < 0.01 and ^@@@^ *p* < 0.001 vs. TFS group; ^&^ *p* < 0.05, ^&&^ *p* < 0.01 and ^&&&^ *p* < 0.001 vs. K + TFS group. ANOVA test with Tukey’s multiple comparisons test.

Animals of the TFS group did not present any behavioral or EEG alterations during the daily TFS. Elisa assay revealed that this group presented similar P-gp protein concentration in all structures analyzed (50.31 ± 15.5 and 54.22 ± 6.78 ng/mL, right and left hippocampi, 60.55 ± 8.88 and 59.75 ± 10.69 ng/mL, right and left neocortex, respectively) compared to the naïve group(*p* > 0.05) (Figure 4).

The ADT for the Kindled group was 356 ± 73 µA and 15 ± 3.1 kindling trials were necessary to achieve the kindled stage (Figure 3). During the last stage V seizure, the amygdala afterdischarge lasted 88.27 ± 18.16 s and its frequency was 3.04 ± 0.26 spikes per second. The afterdischarge propagated to the cortex and lasted 70.11 ± 34.1 with a frequency of 2.2 ± 0.26. Elisa assay revealed a 132% increase in the hippocampus ipsilateral to the amygdala stimulated (111.6 ± 27.1, *p* < 0.001 vs. naïve and TFS groups), 138% increase in the contralateral hippocampus (95.31 ± 24.17, *p* < 0.001 vs. naïve and TFS groups), 51% in the ipsilateral neocortex (99.92 ± 32.63, *p* > 0.05 vs. naïve group and *p* < 0.05 vs. TFS group), and 92% in the contralateral neocortex (97.3 ± 13.76, *p* < 0.001 vs. naïve group and *p* < 0.01 vs. TFS group) (Figure 4).

**Figure 5:**
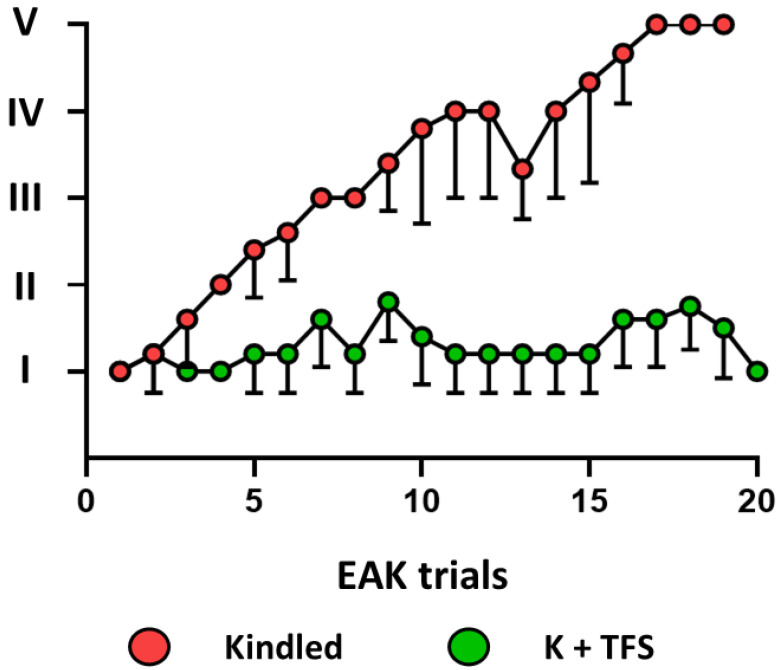
TFS applied immediately after each EAK trial interfered with kindling progression. Graph represents the mean—standard deviation of the behavioral seizure evoked by each daily kindling trial in the kindled and K + TFS groups.

The ADT for the K + TFS group was 284 ± 71 µA (*p* > 0.05 vs. kindled). During the experimental procedure, K + TFS trials only evoked stage I and stage II seizures (Figure 5). During the last stimulation, the amygdala afterdischarge lasted 6.14 ± 4.6 s (*p* < 0.001 vs. kindled group last seizure) and its frequency was 1.6 ± 0.46 spikes per second (*p* < 0.001 vs. kindled group last seizure). Cortical propagation of the afterdischarge was absent in this group. Elisa assay revealed P-gp protein concentration remained like those obtained for the naïve group in both hippocampi (0.39% and 6%, 48.24 ± 9.97 and 52.94 ± 3.32, ipsi- and contralateral to the amygdala stimulated respectively, *p* > 0.05 vs. naïve and *p* < 0.001 vs. kindled group) and both neocortices (−0.7% and 3%, 62.73 ± 8.17 and 62.29 ± 12.7, ipsi- and contralateral respectively, *p* > 0.05 vs. naïve and *p* < 0.05 vs. kindled group) (Figure 4).

## 4. Discussion

Our experiments revealed that EAK-induced epileptogenesis is associated with progressive increase in P-gp expression. These changes are structure-specific and dependent on seizure severity. On the other hand, the antiepileptogenic effect induced by TFS during the EAK is associated with inhibition of P-gp overexpression.

**Figure 6:**
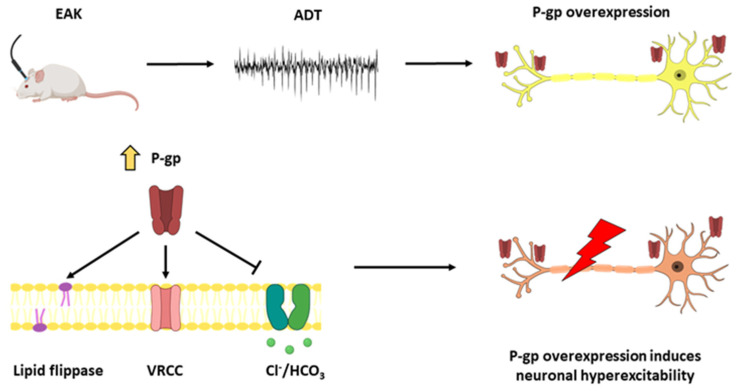
ADT determination induces P-gp overexpression and is associated with epileptogenesis. ADT determination induces P-gp overexpression in the ipsilateral hippocampus. P-gp overexpression induces neuronal hyperexcitability through diverse mechanisms and facilitates epileptogenesis. ADT: afterdischarge threshold. EAK: electrical amygdala kindling. P-gp: P-glycoprotein. VRCC: volume regulated chloride channels.

P-gp overexpression has been found in brain tissue resected from patients with pharmacoresistant epilepsy [12,15,16,18]. In these patients, P-gp overexpressed at the BBB would limit the penetration of antiseizure mediations to the brain parenchyma, reducing their therapeutic efficacy and thus, facilitating the pharmacoresistant phenotype [56,57,58]. Evidence suggests that a single convulsive seizure may induce P-gp overexpression in the hippocampus and neocortex of the rat [38]. The results of the present study indicate for the first time that P-gp is overexpressed during EAK progression. This alteration is present since the first afterdischarge and depends on the brain area involved in the process and the rate of epileptogenesis. This data reveal that first, P-gp overexpression is not a phenomenon restricted to pharmacoresistant epilepsy and second, P-gp overexpression found after a seizure activity may be associated to local changes in network susceptibility (Figure 6).

P-gp overexpression may be induced by several mechanisms including hypoxia [19,20], neuroinflammation [21,22], and/or excessive glutamate release [22,23], all conditions associated with seizure activity [24,25,26,27,28].

An interesting finding from the present study was that the ADT estimation in naïve animals resulted in P-gp overexpression in the hippocampus ipsilateral to the stimulation. Previous data showed that EAK stages 0–I seizures propagated to the ipsilateral hippocampus even in a previously healthy network [48,59]. In addition, ipsilateral structures of the limbic system showed hypometabolism during EAK stages 0–I [59]. Glucose hypometabolism is recently considered a biomarker of increased glycogen consumption, high metabolic demand, and neuronal activity [60,61,62]. This group of evidence suggests that P-gp overexpression at EAK stages 0–1 is the consequence of an environment favoring hyperexcitability characterized by increased extracellular glutamate, K+, and oxygen reactive species [63,64,65]. Supporting this notion, it is known that the systemic administration of a single subconvulsant dose of pentylenetetrazol results in increased glutamate release in the amygdala [66]. However, further studies are necessary to support this hypothesis and the participation of hypoxia or neuroinflammation in the P-gp overexpression at early kindling stages.

In the present study, the expression of the first stage III seizure was associated with P-gp overexpression in both hippocampi. Previous experiments showed that EAK stage III seizures appeared when the afterdischarge propagated to the contralateral hippocampus [48], an effect associated with an increased glutamate release in the ipsilateral hippocampus [67]. Interestingly, hypometabolism was present 24 h after the first stage III seizures in the contralateral hippocampus [59], suggesting an increased neuronal activity, decreased glucose metabolism, and a shift toward glycogen consumption in response to its recruitment. Concerning fully kindled rats, P-gp was overexpressed in hippocampus and neocortex, ipsi- and contralateral to the stimulation. P-gp overexpression in neocortex of fully kindled animals could be explained as a consequence of its recruitment during stage V seizures [46,68]. Indeed, stage V seizures are associated with increased glutamate release in the amygdala and hippocampus during interictal and ictal periods [67,69,70,71] and increased connectivity between the amygdala and cortices [46,68].

P-gp overexpression in neurons has been associated with hyperexcitability and thus, facilitates epileptogenesis [72]. This hypothesis originates from the findings that P-gp can alter the resting membrane potential of neurons as a consequence of changes in the volume-gated chloride channels, the cellular pH, and acting as a lipid flippase [41,42,43,44,45]. Our data show for the first time that P-gp is overexpressed during the EAK in brain areas related to seizure propagation, which may be facilitating hyperexcitability and epileptogenesis. An important limitation of the present study is that we did not assess P-gp expression in cellular types. Other authors did not find P-gp expression in brain parenchymal cells of kindled rats [73,74]. This situation can be explained as the consequence of different times of evaluation, antibodies specificity, and the protocol variables [75]. Future studies are necessary to assess if EAK induces neuronal P-gp expression. 

On the other hand, our experiments support previous studies indicating that TFS applied immediately after each EAK trial interfered with kindling progression, delaying epileptogenesis [40]. In the present study, TFS current intensity was estimated using the electric field that induced the antiepileptogenic effect in cats [40] and translated to the rat brain limbic regions [29] without inducing tissue damage [30,32]. With this assessment, we expected that TFS applied over the skull of the rat generates an electric field that reaches subcortical regions involved in seizure propagation. As such, the antiepileptogenic effect we found in the rat was like that obtained by Valdés-Cruz and colleagues [40] who reported that TFS applied immediately after each EAK trial interfered with kindling progression in the cat.

The mechanisms of the antiepileptogenic effect of TFS are unknown. However, kindling progression is associated with increased glutamate release and activation of NMDA receptors [66,67,76,77]. Interestingly, there is evidence showing that TFS applied during pilocarpine-induced status epilepticus reversed the increased glutamate release in the hippocampus [39]. Another mechanism that may be involved in TFS antiepileptogenic effect is the conduction block effect. This effect, induced during high frequency stimulation (>50 Hz), results in desynchronization of action potentials, increase in the axonal refractory period and potassium overload [78,79]. Lastly, preliminary results show that TFS increased the expression of NSF and decreased the expression of Sema3b in the cerebral cortex and increased Acsm5 and Cml3 in the hippocampus of naïve rats [80]. Future studies are needed to assess if the antiepileptogenic effect is associated with increased modulation of signaling cascades of neuroplasticity or decreased modulation of neuroinflammation and/or glutamatergic transmission.

As for other types of neuromodulations, low frequency stimulation (LFS) applied immediately after each kindling trial interfered with kindling progression [81,82]. Interestingly, delaying LFS did not present the antiepileptogenic effect [82]. Lastly, Wang and colleagues found that LFS delivered after the rat achieved stages II or III seizures did not modify the kindling progression and even worsened the seizure expression. Paired with the results of multielectrode arrays and microPET (micro positron emission tomography), the authors conclude that the limbic system formed an early focal network of afterdischarge propagation during the early stages of EAK [59]. Similarly, Valdés-Cruz and colleagues described that TFS applied before each kindling trial did not modify the kindling evolution in cats [40]. Our results support that TFS-induced antiepileptogenic effects rely on its application during the afterdischarge or epileptiform activity. We suggest that TFS antiepileptogenic effect could be associated with modulation of neuronal activity in the early focal network, avoiding P-gp overexpression and abolishing the recruitment of extra-limbic structures. 

Studies evaluating human brain tissue support that higher P-gp activity is associated with neuronal hyperexcitability [83]. A recent publication from our group described that patients with posttraumatic epilepsy of long duration and poor surgical outcome had increased P-gp neuronal expression. Similarly, patients with frontal lobe epilepsy as consequence of tumors with shorter duration presented neuronal P-gp overexpression [16]. This study suggests that neuronal P-gp overexpression is present since the early stages of the disease and could be favoring epileptogenesis. Further studies using PET imaging are essential to determine the impact of P-gp overexpression in subjects with high risk of developing epilepsy. It is important to notice that P-gp overexpression in the brain has been considered as a therapeutic target to control drug-resistant epilepsy in humans. In this regard, Elkhayat and colleagues did not find changes in the seizure frequency of patients with drug-resistant epilepsy administered with verapamil (1.5 mg/kg/d), a P-gp inhibitor. The lack of efficacy inhibiting P-gp was explained by the long course of intractability and the persistent severe seizures [84]. Interestingly, Asadi-Pooya and colleagues found that adding higher doses of verapamil (120 mg/kg and 240 mg/kg) to the antiseizure medication resulted in an increased seizure control in a dose-dependent manner in patients with drug-resistant epilepsy [85]. Despite these positive results, verapamil induces adverse cardiovascular effects that limit its use as adjuvant treatment in the control of drug-resistant epilepsy. In this regard, TFS represents a non-invasive therapeutic approach to avoid epileptogenesis. Future studies should be designed to assess the optimal parameters of TFS necessary to suppress P-gp overexpression in human brain areas involved in seizure propagation.

## 5. Conclusions

Our experiments revealed that: (1) EAK-induced epileptogenesis is associated with progressive increase of P-gp expression. These changes are structure-specific and dependent on seizure severity. (2) inhibition of P-gp overexpression during epileptogenesis was associated with the antiepileptogenic effect of TFS. These findings are promising as TFS represents a novel therapeutic alternative of non-invasive neuromodulation in patients with high risk of epileptogenesis. Future studies should be performed in sight of clinical application.

## Figures and Tables

**Table 1:** Results of the correlation analysis between data obtained during EAK and the P-gp relative expression to actin in ipsi- and contralateral structures of the kindled group. Freq: frequency (spikes per second), Dur: duration of the afterdischarge (seconds). Values represent the Spearman R. Bold number indicates *p* < 0.05.

Site	P-gp Expression	Kindling Trials	Stage I		Stage II		Stage III		Stage IV		Stage V	
			Freq	Dur	Freq	Dur	Freq	Dur	Freq	Dur	Freq	Dur
Ipsilateral	Hippocampus	−0.791	−0.1	0.8	−0.4	0.6	0.3	−0.3	−0.5	−0.8	−0.4	−0.5
	Cortex	−0.316	−0.7	0.1	−0.4	0.6	−0.6	−0.4	−0.6	−0.6	0.5	−0.1
Contralateral	Hippocampus	0.316	−0.8	−0.6	−0.8	−0.2	−0.9	−0.1	0.1	0.1	**0.96**	0.6
	Cortex	0	−0.6	−0.2	−0.4	0.6	−0.7	−0.3	−0.5	−0.3	0.6	0

## Data Availability

Data is available upon request.
